# Therapy Dog Welfare Revisited: A Review of the Literature

**DOI:** 10.3390/vetsci8100226

**Published:** 2021-10-12

**Authors:** Lisa Maria Glenk, Sandra Foltin

**Affiliations:** 1 Comparative Medicine, The Interuniversity Messerli Research Institute of the University of Veterinary Medicine Vienna, Medical University Vienna and University Vienna, 1210 Vienna, Austria; 2 Department of Biology, University of Duisburg-Essen, 45141 Essen, Germany; sfoltin@web.de

**Keywords:** dog, canine, therapy, animal-assisted intervention, welfare, stress

## Abstract

During the past decade, the field of human–animal interaction(s) research has been characterized by a significant increase in scientific findings. These data have contributed to our current understanding of how humans may benefit from contact with animals. However, the animal experience of these interactions is still an under-researched area. This paper addresses the welfare of dogs who participate in animal-assisted interventions (AAIs) to improve health in human recipients. This paper builds on previous work by Glenk (2017) and provides an updated review of the literature on therapy dog welfare published from 2017–2021. New advances in scientific methodology, such as the determination of salivary oxytocin, breath rate and tympanic membrane temperature, are analyzed regarding their value and limitations for research in AAIs. Moreover, welfare-related social and environmental factors (e.g., freedom of choice, exploration of novel environments, inequity aversion, individual development, working experience, relationship with handler and handler skills) that profoundly influence dog perception and well-being are reviewed and discussed. Accounting for the globally increasing interest and the number of dogs utilized in AAIs, safeguarding therapy dog well-being, and identifying situations, circumstances and protocols that may challenge animal welfare remains an emerging and crucial area of scientific effort.

## 1. Introduction

Animal-assisted interventions (AAIs) seek to positively affect human health by utilizing animals as adjuncts to therapy. AAIs are broadly defined as any practice that involves animals as a part of a therapeutic or ameliorative process [1]. In an AAI setting, the intensity and duration of the procedure vary with the recipient′s particular situation-specific need [2]. Dogs are the species most often involved in AAIs as they are convenient to work with, can be easily trained, and are widely available. Hediger et al. [3] and Menna et al. [4] propose a One Health framework for AAI research outlining under which circumstances no tradeoff of human benefits against animal health and well-being ensue and under which circumstances animals could benefit from such interactions [3,4]. However, few studies measured the effects on the dogs involved [2]. The One Health concept recognizes that human and animal health, as well as their environment, are interconnected. From a One Health perspective, ethically justifiable AAIs should generate added value in health and well-being for all participants, humans as well as non-human animals. During the last few years, One Health has been understood to be an important framework for AAI which entails benefits and risks in the participation for humans and animals that need to be identified [3,4,5]. Enrichment behavior and welfare should, thus, be maximized and distress and health risks minimized, both intra- and interspecifically. Additionally, the triadic interrelationship should be considered and a parallel research design is needed that focuses on and investigates the enhancement of positive welfare indicators using multidimensional and systemic approaches. The One Health approach aims to minimize the tradeoff between human and animal health issues and future research may reveal synergistic benefits of the inextricable linkage within the diverse settings. To achieve added value, interdependency between interspecific health issues should be considered to ascertain that all species enjoy the AAI activity.

Although “partners” in AAIs, humans and dogs do not share equal privilege or power and it is questionable whether dogs give any form of free and informed consent to fulfill the tasks assigned to them. In this Dyad (or rather, triad) dogs must be viewed as the least controlling participant. Within the AAI setting, dogs are often not perceived as animals which we objectify or instrumentalize as the term “partner” indicates some mutual relationship rather than an exploitative one. Current perspectives on AAI animal status are changing in that they should not be seen as “less than” or “tools” but as individuals with likes, dislikes, and limitations [6]. The rationale for this paradigm alteration is practical and ethical since animal welfare is interconnected with recipient welfare. AAI handlers must recognize the power-differential within this triadic relationship and aim to protect their dog′s optimal welfare despite the recipient′s or handler’s preferences. Awareness of, and ability to negotiate, the multiple relationships that are inherent in AAIs should be reflected by AAI handlers attempting to give their dogs a choice at all times to opt-out of participation [7]. Each dog′s voluntariness and suitability should be evaluated objectively on a continuous, ongoing basis, including the use of positive training methods to foster learning without pain, fear, or coercion. Pet Partners, for instance, promote the position that as sentient beings, animals can communicate their “preferred level of participation in AAI” and should enjoy participation in AAIs. The practice of animals merely tolerating the interaction and following the handler′s commands is explicitly criticized [6,8]. The Animal Assisted Intervention International Standards (2019) applies the Five Domains Model [9] (p. 4) expanding the “Five Freedoms” by providing an operational methodology for evaluating conditions impacting animal welfare and affecting the quality of the animal′s mental state. The aims stated are, for instance, to “Minimize breathlessness, nausea, pain and other aversive experiences and promote the pleasures of robustness, vigor, strength and coordinated physical activity” [10] (p. 2) and to “Promote various forms of comfort, pleasure, interest, confidence and a sense of control” [10] (p. 2). Thus, they embrace more than an ideal or aspirational state or principle and offer a guideline for animal welfare assessment and management. The protection of the dog′s welfare and well-being are defined by standards for temperament, training, preparation, socialization and assessment of the dog(s). The importance of the dog′s subjective experience is emphasized. Notably, the AAII′s Standards of Practice state that animals are “sentient beings with needs equal to that of the person” [9] (p. 18).

Adding a sentient being to a therapeutic setting, therefore, creates a triangular relationship rather than a bidirectional one. Having an additional sentient partner requires the adept skills of the AAI handler in order to split attention, maintain desired outcomes, and respond quickly and appropriately to all participants throughout a session. If we support the concept of animals as sentient beings whose rights and dignity may be inseparable from our own, then the ethical principles of autonomy, beneficence, nonmaleficence, and justice should be applied [11]. Respect for autonomy is a norm that obliges AAI handlers to respect the decisions, in the sense of self-determination, of his or her dog, including not just an acceptance of certain behavior, preferences, affinities and dislikes but the promotion of the development of their individual autonomy. The principle of nonmaleficence holds that any AAI handler has an obligation not to inflict harm, not to cause pain or suffering, and not to incapacitate (physically or mentally) his or her dog. The principle of beneficence states the AAI handler′s moral obligation to act for the benefit of the animal. This includes the moral rules or responsibilities to protect and defend the rights of the dog; to prevent harm from occurring and to remove conditions that will cause harm to it. The principle of justice obliges the AAI handler to equitably distribute benefits (time, attention, etc.) between the recipient and dog. Thus, a change from passive to proactive commitment to dog welfare would be required.

The aim of this paper is to provide a review of the recent literature on the welfare of therapy dogs participating in AAIs. Moreover, we sought to discuss the research outcomes in the light of further aspects that clearly affect canine well-being but have previously received little attention.

## 2. Results

Inclusion criteria for the reviewed literature were an experimental assessment of dog welfare indicators linked to performance in AAIs and the publication in a peer-reviewed scientific journal. Exclusion criteria included publication before 2017, duplicate publications, publication as a monograph or academic thesis, commentaries, reviews and meta-analyses. Scientific databases, as well as titles and keywords of publications, were screened for: therapy dog, canine, welfare, well-being, stress, arousal, behavior, AAI, animal-assisted therapy (AAT).

### 2.1. Overview on Included Studies

The literature research revealed twelve relevant peer-reviewed papers that were published between 2017 and 2021. As this paper builds on a previous review of the literature [2] duplicate publications were identified and not considered in the present analysis, resulting in the exclusion of one study [12].

In all of the studies, dogs participated in AAIs that were organized as visitation programs (Table 1). The AAI sessions were carried out in different environments such as a hospital, healthcare or outpatient unit, kindergarten, school, university campus, prison, and training campsite of the organization. Dog welfare indicators were salivary cortisol, behavior, heart rate, heart rate variability, respiratory rate, salivary oxytocin, tympanic ear membrane temperature and observed stress levels. Evaluations were based on self-reports of dog handlers, recipients and the experimenter. As depicted in Table 2, session duration varied between ten and ninety minutes, intervals between sessions ranged from 15 min to once per month; however, four studies did not provide any further information. Six studies included adult recipients, four studies included child recipients, and two studies did not provide any further information. Seven studies included programs in which recipients participated in groups of two or more individuals. Two studies included programs with single recipient sessions, two studies included both single and group sessions, and one study did not provide any further information. The number of dogs included in the studies ranged from two to forty individuals (Md = 7.5, SD = 11.45).

### 2.2. Description of Study Outcomes

Pirrone et al. [13] studied social synchronization patterns in handlers and dogs. Moreover, the assessment of heart rate and salivary cortisol was carried out over the course of five subsequent AAA sessions with psychologically or physiologically disabled adults. Gaze synchrony, joint attention and touch synchrony were registered before, during, and after the sessions. Social synchrony occurred prior to and during AAAs with joint attention being the most prevalent behavior. However, more gaze synchrony and joint attention were found during AAA performances than prior to sessions. No differences in salivary cortisol levels were found except for individual differences between the dogs. Although heart rate was higher in dogs on working days with AAA sessions compared to control days, values remained within the common physiological range, suggesting only minor increased arousal. Individual preferences for physical contact with recipients were described with some dogs being more willing to initiate physical contact with the recipients than others [13].

McCullough et al. [14] published their findings on salivary cortisol and behavior in dogs performing AAIs in pediatric oncology. Sessions were arranged so that a dog-handler team was paired with a child, his or her parents and hospital staff. No significant differences in the dogs′ salivary cortisol were detected when working concentrations were compared to pre-working levels at the hospital site or at home. However, during AAI sessions, higher salivary cortisol was associated with an increased frequency of stress behaviors and a reduced frequency of affiliative behaviors. In dogs that exhibited higher scores of stranger-directed fear in a behavior-centered questionnaire (i.e., C-BARQ), fewer affiliative behaviors were displayed in AAI sessions. The findings suggest that only mild expressions of distress in dogs were observed; however, notably, incidences of stress and affiliative behaviors were linked with certain activities. For instance, more stress-related behaviors were seen if the child put a bandanna on the dog and fewer affiliative behaviors were found if the child used a stethoscope to listen to the dog′s heartbeat, the child played a game on the dog′s vest or drew a picture of the dog [14].

Colussi et al. [15] carried out an exploratory study on dog salivary cortisol responsiveness during various cognitive and physical activities that included AAA as the stimulus. In their study, dogs participated with their owners in group interventions in kindergarten, where children had verbal and tactile contact with the dogs. To assess working concentrations of cortisol, a pre-session saliva specimen was collected and compared to a post-session sample. In addition, home baseline samples were gathered. The results on working and resting salivary cortisol found no AAI-related increase. The authors stated that AAAs can be considered as low-intensity exercises, while dogs provide high psychological support to recipients. Significantly higher pre-session cortisol levels may be associated with anticipation stress or arousal during transportation to the facility, but a causal relationship could not be inferred [15].

Uccheddu et al. [16] presented a case study on two dogs participating in weekly animal-assisted reading sessions for children with developmental disorders. In the reading interventions, the therapy dogs interacted for 30 min with a group of five children. Dog welfare indicators were salivary cortisol and behavior. One of the dogs had higher salivary cortisol responses pre-session and during the session compared to the other dog and the control condition. The most frequently observed stress behaviors were tail down and lip licking. The authors linked salivary cortisol reactions and behaviors to transportation distress or session anticipation. In general, Uccheddu et al. (2018) concluded that participation in the reading program did not result in any significant changes in physiological or behavioral variables [16].

Corsetti et al. [17] performed a behavioral study where therapy dogs participated in single AAI sessions with recipients who had mental or psychomotor disabilities. The sessions were held outdoors and indoors and were carried out at the training campsite of the AAI organization or in hospital environments. There was no difference in the occurrence of canine anxiety-related or submissive behavior during AAIs. However, the sessions were marked by an increase in non-aggressive dominance behavior, attention, sniffing, affiliative and playful behavior. Dogs were more attentive to their handlers compared to recipients or other people present. Accounting for these findings, the authors considered the workload the therapy dogs were exposed to during their investigation as adequate [17].

Clark et al. [18] studied pre- and post-session salivary cortisol responses of four therapy dogs visiting outpatient nursing units in varying intervals between sessions (i.e., biweekly, weekly, once, and twice a month). This study was the first to assess the frequencies of visits with regard to therapy dog physiological welfare. The results suggest a decrease in salivary cortisol when dogs visited the facility twice a week. Moreover, the youngest and least experienced dog of the sample was seven years old and had higher post-session cortisol values than her conspecifics in all conditions except the weekly interval scenario. In contrast, the oldest dog (twelve years old) had the lowest post-session cortisol values in all conditions except the biweekly interval scenario [18].

Silas et al. [19] examined the effect of therapy dogs participating in on-campus AAI programs. Foci were the prevalence of stress behaviors in therapy dogs, their handlers, and student AAI recipients. The research instrument was a 5-point, Likert-type visual analog scale. While there was a general reduction from pre- to post-session in the stress perception of both therapy dog handlers and students, the therapy dog experience was marked by an increase in stress when home baseline ratings were compared to post-session ratings. Interestingly, self-reported handler (not recipient) stress levels were related to more stress behaviors in their dog. According to handler ratings, 25% of therapy dogs had increased stress levels, 22.5% had decreased stress levels, and 52.5% had no change in stress level over the course of their time working in a session. The authors explain their findings with a certain excitement arising in therapy dogs when they are taken to a novel environment. The study also revealed some inconsistencies when correlating handler and researcher stress level ratings, suggesting that handler perceptions may be more accurate in reflecting their dogs′ experiences [19].

Research findings by Melco et al. [20] do not suggest that therapy dogs were hyper-aroused by participating in repeated animal-assisted therapy sessions with a group of three to four children with an attention deficit hyperactivity disorder (ADHD) diagnosis. The interventions were structured into calming activities where dogs both quietly interacted with their owners and performed therapeutic exercises with the children. Neither the dogs′ salivary cortisol levels nor heart rates showed any changes associated with therapy performance across six sessions. The study outcomes point at only mild stress that was evident in increased stress behaviors such as ears back, panting and lip licking [20].

One study documented an increase in salivary cortisol, heart rate and respiratory rate in experienced dogs when their AAI-related values were compared with baseline measurements at home [21]. The dataset included a large variety of AAI settings regarding the environment, recipients, session duration and frequency. Outcomes of the study did not reveal any associations with AAI-related settings such as session duration and frequencies, time of day, recipient, dog or recipient characteristics, or whether dogs were handled with or without a leash. However, dogs performing AAA had higher heart rates than dogs who were involved in AAT. In addition, dogs that had to be transported for more than 50 min from their homes to the AAI sites panted more and had higher heart rates. Some dogs provided values considered outside of physiological thresholds, which were attributed to high temperature, fear of being transported by car and being overcrowded by recipients [21].

Clark et al. [22] collected pre- and post-session salivary cortisol samples from nine dogs and their handlers performing AAIs in a hospital. Handlers also completed a survey on their own and their dog′s perceived stress levels, which was complemented by behavioral observations. No differences were found in dog or handler salivary cortisol when baseline values at home were compared to working levels across multiple measurements. However, a significant correlation between the owner′s perceived stress in the dog and dog salivary cortisol was evident. These findings suggest that the handlers were sensitive to their dog’s experiences and that their perception is in line with the physiological state of their dog. The most frequently observed stress behaviors were panting, lip licking and yawning [22].

In another recent study, Clark et al. [23] used multiple parameters to assess the emotional state in therapy dogs including salivary cortisol, oxytocin, tympanic ear membrane temperature, heart rate and heart rate variability as indicators of well-being. Nineteen dogs participated in both single recipient and group therapy sessions with patients diagnosed with fibromyalgia. Over the course of five sessions, no changes in cortisol or oxytocin emerged; heart rates and right tympanic ear membrane temperatures were significantly lower post-session, indicating a neutral to emotionally positive reaction towards the sessions. These novel findings indicate dogs were possibly in a more relaxed state at the end of the AAI session [23].

There was only one study that included kennel-housed shelter dogs participating in AAIs [24]. For the purpose of the study, a veterinary behavior expert pre-selected well-suited candidates based on canine personality and behavior from a shelter dog population. For the AAIs, dogs were transported from the shelter to a prison, where they interacted weekly with two inmates for a period of two months. The research findings revealed a significant decrease in baseline cortisol levels at the end of the AAI program, indicating a decline in physiological arousal. In addition, transportation for approximately 60 min resulted in a significant increase of the dogs′ cortisol levels compared to baseline measurements at the kennel or post AAI session. The study results highlight that structured interaction with humans outside the shelter environment seems to be efficient in buffering hyperarousal linked with high cortisol levels in kennel-housed dogs [24].

## 3. Discussion

The past years have been marked by an increase in scientific interest in the canine perspective of AAIs. Research methods are similar to those described by Glenk [2], thus, the analysis of salivary cortisol, behavior and perceived stress level evaluations continue to be the most common approaches in assessing dog welfare. Novel methods include the determination of salivary oxytocin, tympanic ear membrane temperature and respiration rates. Research outcomes indicating that working salivary cortisol concentrations are similar to baseline measurements gathered on non-working days [13,14,15,20,22] parallel previous data from Glenk et al. [25,26], Ng et al. [27] and Koda et al. [28]. A study with shelter dogs reported a significant decrease in basal cortisol concentrations across the two months of a weekly AAI program [24]. In contrast, studies have found an AAI-related increase in behavioral stress [19], salivary cortisol [21], heart rate [13,21] and breath rate [21]). These outcomes reflect earlier data by Haubenhofer and Kirchengast [29,30] and King et al. [31]. Although indicators of welfare appeared to be significantly raised, the authors concluded that they predominantly fluctuate within the physiological range. However, it emerged that increased canine stress behaviors [14] and perceived canine stress levels rated by the handler [22] were positively correlated with salivary cortisol. In addition, Silas et al. [19] found that canine stress levels were positively associated with handler self-reported stress. However, therapy dogs’ post-session cortisol levels were lower in dogs rated as minimally stressed but there was no change in salivary cortisol in dogs that were rated as severely stressed by their handlers [28]. In sum, these data suggest that AAI performances are sometimes linked with higher arousal and that handler perceptions can reflect the dog experience to some extent. Participation in an AAI session stimulated both synchronous behaviors with the owner [13] and socio-positive behaviors such as attention, sniffing, affiliative and playful behavior [17]. Lying under command while surrounded by multiple recipients led to an increase in salivary cortisol in one dog [21]. Restraint has previously been linked with higher arousal that increase urinary adrenaline and noradrenaline [32].

Additionally, dogs displayed more stress-related and fewer affiliate behaviors if recipients carried out actions directly on the dog [14]. These findings underline the importance of recipient instruction prior to any human–animal interaction as both recipients and staff members may not automatically interact appropriately with the therapy dog [33]. Accordingly, briefing recipients and personnel on canine species-specific and individual needs is essential for safeguarding dog welfare. An issue that was raised in several publications was transportation. Transport duration of between 50 and 60 min were related to increased physiological arousal [21,24]. Caution is warranted if dogs experience transportation distress such as fear of car rides [15,16,21]. Eight out of twelve studies had dog sample sizes with less than ten individuals. The literature review revealed a median of 7.5 dogs, thus, the science on therapy dog welfare is still characterized by relatively small samples of included dogs. Although Iannuzzi and Rowan already stated in 1991, that AAI sessions should be no longer than 60 min, in three recent studies [15,18,24] dogs participated in sessions between 70 and 90 min [34].

### 3.1. Relationship

In their article ‘The Three Rs as a Framework for Considering the Ethics of Animal Assisted Interventions′, Simonato et al. [35] contextualized AAIs with regard to ethical concerns for the animals involved and proposed an adaptation of the Three Rs principle (replacement, reduction, refinement) [36]. According to the nature of AAIs, animals are incorporated in a heterospecies interactive process as agents to improve human recipient motivation, health and/or well-being. Although Marino [37] has challenged this concept, due to the lack of scientific evidence as to whether and to what extent a living animal is the indispensable ingredient in AAIs, a plethora of studies have sought to substantiate the effectiveness of AAIs based on the theory that the animal is the essential component [37].

Is it feasible to replace the animal in an AAI? An emerging body of literature has stated that robot animals may work as substitutes for living animals to some extent [38]. While the benefits of robot animals in terms of their compatibility regarding animal welfare and hygiene are unquestionable, their effectiveness in affecting human health outcomes and emotional states has been questioned. Biomimetic Robotic Dogs, while not a topic within this review, have been discussed in a number of recent publications. Barber et al. found that robotic dogs may be just as effective and a suitable replacement in certain situations with therapeutic benefits for children and young people [39]. The children spent a comparable amount of time stroking both the real-life dog and the robot and spent more time interacting with the robot, even though the children reported they significantly preferred the sessions with the living dog. Bradwell et al. pointed out the importance of user-centered design opinion on desirable features of companion robots as contradictory research outcomes suggest robot design is not always optimal for the end-user, in this case, geriatric patients [40]. Romano et al. focused, in their review, on an animal–robot interactive setting, thereby merging the biological and artificial world on a physical and cognitive level [41]. Aarskog et al. provided a systematic overview of the effects of animal-assisted intervention (AAI) using either dogs or robotic animals [42]. Both reduced behavioral and psychological symptoms and depression and improved mood and quality of life among residents with dementia in nursing homes. This has been further extrapolated in the systematic review and meta-analysis of Ong et al. [43]. Thus, this field of development warrants further research and discussion.

Therefore, in line with the Three Rs, Simonato et al. [35] proposed to not absolutely, but to relatively replace, animals in AAIs by choosing the appropriate species and the most appropriate individuals within that species. The preferred choice should be based on the animal′s ethogram, individual characteristics, training status, the aim of the project, the type of setting, and the recipient(s) involved. In optimizing the working conditions for animals in AAI, a reduction in the number of participating animals and recipients as well as the number of sessions and the duration appears feasible. Regarding the refinement of an AAI, Simonato et al. [35] highlight the importance of controlling for ideal environmental conditions, proper animal care and best practice for educating and selecting people who are involved. Finally, the relationship between human and animal has been suggested as a fourth R dimension, a non-instrumental relation in which species-specific and individual needs are well-taken care of [35]. In fact, the human–dog dyad has been characterized as a mutual relationship affecting both physiology and mental state [44]. An attachment refers to an intense, emotional relationship between two individuals [45]. Several studies have sought to investigate dog–human relationship characteristics and compare them to human caregiver–infant relationships by introducing an experimental test paradigm [46,47,48]. Thus, attachment has also been associated with the human–dog dyad and is characterized by behaviors like proximity seeking, exploration and separation distress. Moreover, stressful experiences may be buffered by the support of the human attachment figure and different attachment styles are also prevalent in dogs [49]. Recent data by Wanser and Udell [50] add to these findings suggesting that the behavior of securely and insecurely attached dogs participating in laboratory-simulated animal-assisted activities did not differ. However, dogs spent significantly more time in proximity to and touching the recipient while gazing more at their handlers. Thus, the handler′s presence may serve as a secure base for dogs to confidently perform the AAA-related tasks and dogs may use their gaze during social referencing. Wanser and Udell also reported that insecurely attached dogs tended to gaze more often at their handlers [50]. In fact, frequent gazing at the owner in less secure dogs could be caused by the fact that they require more guidance and reassurance from their handlers. Similar to attachment, behavioral synchrony (i.e., coordinated behaviors reflecting mutual attunement between interacting individuals [51]) is also rooted in the caregiver–infant bond. Although the findings by Pirrone et al. did not reveal any synchronic patterns in the physiological reference markers heart rate or salivary cortisol [13], research in equine-assisted therapy has indicated that synchrony in heart rate varies with the relationship intensity in AAI recipients, horses and therapist [52]. Since cascading levels of stress may decrease synchrony between interacting individuals [53], it is an interesting phenomenon to study in the context of AAIs.

### 3.2. Inequity Aversion

In some AAI settings, several human–dog teams participate simultaneously, sometimes in close proximity to each other. In such cases, handlers should be aware that dogs are sensitive to unequal treatment and may cooperate less if they observe that a conspecific may receive more treats, praise or attention for similar compliance. Inequity aversion has been referred to as human resistance to inequitable treatment [54]. It is suggested to be an adaptive mechanism to promote cooperative interactions between non-related individuals [55]. Inequity aversion research in animals has been initiated by Brosnan and De Waal [56,57]. In their experiments, they investigated the conditions under which capuchin monkeys and chimpanzees were willing to exchange a token with the experimenter for a food reward. Their findings illustrate that the animals refused to collaborate if they watched a conspecific obtain a more desirable food reward for equal or even less effort [56,57]. A similar experimental set-up was used by Range et al. to determine whether dogs are also sensitive to equal reward [58]. The data highlighted that unequally rewarded dogs refused participation in a paw lifting task earlier, hesitated longer to respond to human commands and exhibited more stress-related behaviors [58]. In a follow-up study, Brucks et al. underlined these results by showing that after the experiment, unequally rewarded dogs tended to avoid the experimenter and the conspecific dog in a neutral environment [59]. To date, there are no studies available on inequity aversion in therapy dogs. There is evidence that organizations have screening criteria for volunteer AAI teams including a gentle temperament of the dog and accepting a conspecific [60]. Given the prevalence of inequity aversion in canines, this phenomenon certainly needs to be recognized in AAI practice. Accounting for dog sensitivity toward disadvantageous reward distribution, caution is warranted if multiple dogs participate simultaneously with multiple recipients in an AAI session, which is common in on-campus programs [19,27], in kindergarten [15] or in prison [24,28].

### 3.3. Exploration of AAI Environments

Increased familiarity of the AAI session site and recipient population has been previously linked with lower post-session cortisol [26] or noradrenaline [32] concentrations in therapy dogs. In addition, new data by Clark et al. [18] and Pirrone et al. [13] suggest less arousal when dogs work regularly in the same environment. In the AAI context, exposure to unfamiliar neutral environments has been associated with an increase of cortisol secretion in therapy dogs [27]. This suggests that an adaption phase to help dogs familiarize themselves with a novel environment is essential to support animal welfare. If dogs can function as autonomous agents, environmental exploration will likely generate various forms of comfort, pleasure, interest, confidence and a sense of control. Exploration and the dogs′ resulting basic intellectual capacity affect their ability to produce positive outcomes and prevent negative ones in an AAI setting. When a dog is given the opportunity to explore an environment, predictability is supported, and voluntariness to enter a specific facility or setting is reinforced.

Exploration is an integral part of human and non-human animal cognition and welfare. Cognition is the mental process assisting an organism to acquire, process and use information [61]. Cognition is a cause and consequence of welfare but can later affect cognitive functions [62]. Dogs are motivated to use or enhance their cognitive skills and may suffer when prevented from doing so. Exploration, in the best sense, allows dogs to act autonomously and by their own initiative [63]. It is important for animals to be able to gather information about features of their environment [64] in order to deal with the fear of the unknown. Neophobia, or the fear of novel objects or situations, constrains explorative behavior, and thus, limits opportunities for learning and mental growth. Exploratory behavior is considered an aspect of sensory processing involved in investigating novel stimuli, rather than an instinctive behavior [65] and partially depends on the motor and spatial capabilities and on the motivation to explore [66]. Exploration enhances these capabilities as well, as it enables dogs to problem-solve, to direct and redirect themselves and to analyze their environment by themselves, in other words, to make choices. The retrieval of memorized information is context-dependent i.e., using past experiences for decision-making processes and subsequent behaviors. This signifies the importance of early exploration opportunities being offered to allow the dogs to increase their practice [63]. How a dog seeks support in challenging or new situations is influenced by the type of dog–owner attachment. Fearfulness or anxiety, which inhibits exploration, has been linked to lack of experience and aversive learning strategies [67]. Foltin postulated that an owner who is supporting the dog′s attempts to independently explore leads to more confident, self-reliant dogs displaying cognitive variance and becoming more autonomous [63]. Within AAI handlers, they should provide opportunities for their dogs to behave in ways the dogs find rewarding, thereby enhancing their motivation and enabling them to experience positive welfare states. The Animal Assisted Intervention International Standards (2019) of Practice set forth that the relationship between the human partner and the dog should be one of secure attachment where the dog may rely on their human partner to provide a safe base to obtain their needs and to explore their external environment [9]. Dogs are exposed to frequently changing settings: different rooms, people, as well as temperature, and olfactory and visual information. As a practical recommendation, AAI handlers should be encouraged to offer exploration opportunities to their dog each time they enter a setting. In order to increase control of the situation, heighten autonomy, predictability and structure, dogs should be motivated to explore each setting in their own time. Handlers should not guide them or intervene in their movements or choices. The dogs should be autonomous agents acting on the environment, able to explore unrestrictedly and freely.

### 3.4. Therapy Dog Age

The findings by McCullough et al. [14] and Clark et al. [18] further substantiate the effect of dog age and experience on welfare indicators which was previously reported in a review of the literature by Glenk [2]. However, there are no experimental data available about the ideal age for a dog to participate in therapeutic settings. The welfare of the therapy dog also includes the “after the job” retirement phase. This topic has recently received more attention [68,69,70]. As seen in this review, dogs participate in varying capacities, sometimes in arduous settings based on intense training coupled with high expectations and exposure to the stressors of different environments. Older animals naturally become less resilient, need more time to recover from stress, and thus, may not be as adept at managing social situations [71,72]. Therefore, it is important that dogs can retire at an appropriate point in time from these activities to avoid negative welfare impacts. As retirement components are multifaceted, one must weigh benefits and disadvantages as a sudden sedentary lifestyle may also result in confusion, boredom, frustration, or a lack of physical and mental stimulation for the dogs. Retirement affects not only the animal but the handlers and in cases of long-term relations also the recipient. Challenges may arise, particularly if the handler is not willing to retire his or her dog. However, for ethical, health and liability reasons there must be a necessary phase of retirement. Ideally, the retirement process is carried out gradually and closely supervised by a veterinary behaviorist.

While the benefits of AAIs have been applied across the human lifespan from pediatric to geriatric participants, implications for AAI dogs are not paralleled in research. One problematic area thus far undiscussed is the early exposure of puppies to overly challenging environments i.e., handlers integrating their dogs into AAI settings as soon as they acquire them. When puppies are removed from their mothers as well as their familiar social and physical environment, a particularly sensitive phase is initiated since separation may cause behavioral and physiological distress and augment manifestations of anxiety and depression-like behaviors [73]. Thus, the dogs are already in a highly aroused state. Additionally, puppies are not yet fully developed, physically or mentally, and their social skills are shaped and continuously reinforced by internal and external processes. Evidence indicates that early experiences play an important part in shaping behavior. If carried out appropriately, these experiences may reduce problematic behavior later in life. However, if this process is not well-controlled, it may result in an increased likelihood of undesirable adult behavior [74]. Stimuli and experiences to which puppies are exposed daily in the home are enough to provide them with adequate socialization. From the socialization period (the socialization period of development and maturation of senses lasts until approximately 12–14 weeks according to Scott and Fuller 1965 [75]) and throughout the enrichment period, dog owners should warrant safe exposure to different experiences, objects, animals, and people, while ensuring that the puppy enjoys these experiences and is not alarmed by them [76].

Socialization refers to the process of desensitization, i.e., gradually allowing a puppy to encounter new objects while safeguarding that he or she finds this exposure pleasant. The socialization period is crucial for the development and maintenance of long-term relationships and interactions. In the next period of development, the enrichment period, the dog may then be exposed to more experiences that it will likely encounter in a setting [77] in a step-by-step process, individually adapted to the idiosyncrasies and developmental status of the dog. If puppies are brought to institutions or settings they will be confronted with challenging environments including a variety of novel, unexpected, or potentially aversive stimuli. A diverse range of activities may expose them to high levels of noise, odors, unstable and varied surfaces, etc. In addition to physical factors, dogs may need to cope with a variety of people, some behaving in unpredictable ways. They may also be confronted with other animals and variable routines. Dogs can respond to these types of situations with behavioral signs of fear or anxiety, including freezing, withdrawing, or showing aggression [78]. Early environmental experiences strongly influence the emotional stability of dogs and too much stress can cause pathologic difficulties rather than physical or psychological advantages [79]. Early life is an important time of enhanced synaptic plasticity, where exposure to environmental stimuli has a profound influence on later behavior [80]. The development of the stress system plays a crucial part in shaping its function during adulthood and impacts stress resilience and vulnerability to stress-related disorders later in life [81]. Repeated stressors during the stress-hyperresponsive period produce long-lasting effects on the hippocampal regulation of the hypothalamic–pituitary–adrenocortical axis, which is associated with increased responsiveness to stress stimuli in adulthood [82]. Therefore, puppies are not socially, neurologically, or developmentally mature enough to participate in any AAI setting as they have not yet developed strategies to react to stressful situations [82]. Their natural development includes sensitive periods in which they are particularly fearful and susceptible to various stimuli [78]. Effects of environmental experiences and learning opportunities during this time impact their individual development including their resilience to stress [78].

Little evidence exists as to the optimal timing of stimulus exposure as well as the initial level of exposure. Perhaps composite stimuli need to be broken into their component parts and separately presented, or perhaps puppies may tolerate combined stimuli at low intensity. Because each dog will have unique tolerance to different types of stressors, it is important to select one which is below that to which the dog responds adversely. The benefits of gradual introduction to novel stimuli in contrast to repeated exposure of a full intensity stimulus (“flooding”) need to be understood by therapy dog handlers, trainers and recipients. Currently, there are no standards for best practices in puppy raising techniques. The predictability of the environment is particularly important for the dog, and the handler must, therefore, optimize the method of initial stimulus exposure. Exposing puppies to new situations, such as unaccustomed confinement or social isolation, inconsistent handling and inescapable fear-eliciting situations should be avoided [83].

In practice, many handlers unfortunately do not use this gradual approach. Often a puppy is immediately exposed to unfamiliar locations, in the presence of new people, having been transported in a new vehicle to the location. The presentation of numerous potential stressors simultaneously may lead to sensitization in some individuals [84,85]. Mills et al. identified stressor aspects (affective quality, intensity, magnitude, and duration) and proposed predictability and expectation to decrease their negative impact [86]. The number of potential stressors is significant in AAI settings. Hence, welfare concerns arise from exposing puppies to potentially fear-provoking situations for which they are poorly prepared. Environment affects socialization and it is vitally important to rear the young dogs in optimal settings, introducing them to a range of stimuli in a positive, controlled, and gradual way in order to minimize fears [84]. Therefore, the focus must lie on an appropriate “initiation” process prior to exposing the dogs at an appropriate age, cognitively and emotionally, to any AAI setting. Dog handlers interested in AAIs may certainly enroll in a qualified and quality organization to ensure supervision and peer review and a gradual learning and exposure process accounting for the developmental stage of their puppy. Appropriate education, training and support for trainers and handlers must be offered. Respect for each dog′s age, health, physiology, temperament, ability, and stamina should be guaranteed in any AAI setting and be reflected through applicable guidelines [8]. AAI Organizations should focus to improve and supervise dog handling practices, particularly through promoting guidelines and restricting the practice of puppies being brought into settings.

### 3.5. New Research Methods

The assessment of oxytocin, tympanic ear membrane temperature and respiration are novel methods in AAI research. Previous studies have shown that oxytocin levels rise during affiliate human–dog interactions. This effect has often been accompanied by decreases in cortisol. Oxytocin is a molecule of particular interest given its potency in buffering stress responses by acting on both glucocorticoid and beta-adrenergic receptors. Plasma and salivary oxytocin concentrations correlate [87] but there has been some initial hesitation regarding the feasibility of oxytocin determination in saliva [88]. Recent data suggest that salivary oxytocin may, in fact, better reflect cerebrospinal fluid concentrations than plasma concentrations and, therefore, its scientific use is warranted [89]. Research outcomes from Clark et al. did not reveal any changes in dogs′ oxytocin triggered by human–animal interactions during AAIs in a hospital setting [22]. At first glance, these findings may appear surprising when considering the findings by Odendaal and Meintjes [44], Handlin et al. [90], Nagasawa et al. [91]. There is evidence, however, indicating that an increase in oxytocin does not necessarily equal good welfare. In fact, higher basal oxytocin levels have been related to increased anxiety [92], relationship distress [93] and higher stress vulnerability in humans [94]. In horses, higher basal oxytocin has been observed in individuals that displayed mimic muscle activity associated with discomfort [95]. In piglets, experimental administration of oxytocin has been associated with poor social behavior [96]. These seemingly inconsistent data suggest that more research is needed to further substantiate the causal and relational mechanisms of oxytocin release.

Stress experiences are commonly accompanied by a spontaneous rise in core body temperature. This effect has been widely studied across various mammalian species and has been referred to as stress-induced hyperthermia [97]. Core body temperature can be assessed using infrared thermography [98,99], or like in the study by Clark et al., a tympanic ear thermometer [22]. Acute stress [100] and emotional arousal [101] have been related to changes in right ear tympanic membrane temperature. Accounting for the non-invasive nature of temperature assessment, it should be further applied to research on animals in AAIs. A recent review provides further data on the benefits and challenges of infrared thermography in assessing animal emotional states [102].

The respiratory rate has been used to monitor different pathological conditions and stressors, including emotional stress, cognitive load, heat and cold [103,104]. Emotions affect ventilation and the respiratory rate increases, for instance, with experimentally-induced anticipatory anxiety [103]. The respiratory rate is a marker of emotional stress because it is partially regulated by brain area activities involved in emotional processing [105]. Furthermore, respiration is sensitive to movement and various cognitive tasks, proportionally increasing with the difficulty of a task [106]. Accounting for the fact that panting in dogs is mediated by movement, cognition, emotion and environmental temperature, the interpretation of changes in respiratory rate during AAI sessions remains challenging.

Notably, the variability across the methodology in these studies and the lack of standardized protocols is a limitation and hinders broad comparisons between studies.

## 4. Conclusions and Future Directions

In sum, the current findings support earlier data [2] in that no acute manifestation of distress was reported in any of the studies. If physiological markers of arousal increased, they were predominantly within the physiological range. There is evidence that aspects like transportation, familiarity or frequency of sessions, and age of the dog modulate animal welfare indicators. Some preliminary results suggest that certain types of human–animal interaction are associated with the restricted comfort of the therapy dog including being surrounded, restrained, or if recipients directly carry out actions on the dog. This information should be considered by anyone who designs and provides AAIs. However, there is more insight needed to identify the circumstances under which animals may withdraw from a recipient or the setting.

There still exists a gap in knowledge of the long-term effects on dogs participating in AAI programs. It would be essential to run long-term research including regular, continuous physiological and psychological follow-ups. This would enable us to compare their status at an early stage of their therapy dog career to a later point in time. We have seen that the level of handler arousal is associated with that of the dog and that handlers are able to recognize signs of stress. Furthermore, the quality and intensity of the human–animal bond are essential. These findings open an avenue for future research. Thus, studies may benefit from adding measurements of handler stress coping strategies, emotional competence, attitude and empathy towards their dog. Training methods of the dogs may be incorporated as one important factor as well as more details on training procedures and prior experience. Additionally, it would be interesting to differentiate between different breeds. In most studies, sample sizes are relatively small which hinders the generalization of the findings. Accordingly, future investigations should consider appropriate sample sizes and rigorous methodology to address dog welfare. Further, it must be acknowledged that therapy dog certification procedures vary substantially and so do predictors of suitability for the role of a therapy dog. Future research is challenged to evaluate the feasibility of established certification procedures with regard to the selection of ideal canine candidates.

With regard to puppies, future research should explore innovative methods for best measuring the relative resilience of dogs to stressful events and develop optimal protocols to enhance such resilience [78]. Optimizing welfare necessitates attending to and calibrating the level of cognitive stimulation to the developmental stage of the dogs. Here, a balance point must be found regarding the environmental enrichment and novel input [62]. Future research must also find a way to determine appropriate and individualized parameters and feasible methods to investigate the ideal time for retirement and to prepare the animal handler for such.

What is the ideal baseline? Studies commonly used the home environment or pre-session sample collections as a reference marker for working levels. Regarding pre-session levels, some authors stated that transportation distress or anticipation may have caused physiological or behavioral indicators of discomfort. Thus, the level of arousal pre-session is likely affected by previous experience, the novelty of the environment, and whether the dog is comfortable with transportation. Although multiple studies used the home environment to reference non-working conditions, the feasibility of this procedure has not yet been evaluated. It should be acknowledged that there is enormous variability in the living conditions of each dog. Dogs may live in single-households, traditional families, or patchwork-families, and may be individually-housed or with conspecifics. Accounting for these dynamics, the level of external stimulation and, thus, the physiological baseline of each dog at home may also vary greatly. These factors have not been controlled for in any of the studies, and identification of an ideal baseline remains an unresolved issue that will hopefully be addressed in the future.

## Figures and Tables

**Table 1:** Publication year, therapeutic environment, recipients, sample of dogs and welfare indicators.

	Published in	Environment	Recipients	Dogs (N)	Welfare Indicators
Pirrone et al. [13]	2017	Health care facility	Adults	4	Heart rate, behavior.
McCullough et al. [14]	2018	Pediatric hospital	Children	26	Salivary cortisol, behavior, handler questionnaire.
Colussi et al. [15]	2018	Kindergarten	Children	6	Salivary cortisol.
Uccheddu et al. [16]	2018	Reading program	Children	2	Salivary cortisol, behavior.
Corsetti et al. [17]	2019	Hospital or Training campsite of AAI association	N/a	6	Behavior.
Clark et al. [18]	2019	Outpatient nursing unit	Adults	4	Salivary cortisol.
Silas et al. [19]	2019	University campus	Adults	40	Stress rated in a visual analogue scale.
Melco et al. [20]	2020	Outpatient therapy for children with ADHD	Children	9	Salivary cortisol, heart rate, behavior.
De Carvalho et al. [21]	2020	N/a	N/a	19	Salivary cortisol, heart rate, respiratory rate.
Clark et al. [22]	2020	Hospital	Adults	9	Salivary cortisol, perceived stress protocol, behavior.
Clark et al. [23]	2020	Outpatient clinic	Adults	19	Salivary cortisol and oxytocin, tympanic ear membrane temperature, heart rate and heart rate variability.
D′Angelo et al. [24]	2021	Prison	Inmates	5	Salivary cortisol.

**Table 2:** AAI session characteristics including duration, organization of recipients (single or group sessions), between session intervals and significant findings.

	Duration	Single/ Group	Intervals	Relevant Findings
Pirrone et al. [13]	55 min	Group	Weekly	No difference in salivary cortisol between working and resting days; ↑ salivary cortisol prior to AAA compared to during the activities; ↑ joint attention and gaze synchrony during AAA; ↑ heart rate on working days.
McCullough et al. [14]	20 min	Single	Weekly	No difference in salivary cortisol between working and resting days; ↑ salivary cortisol levels related to ↑ stress behaviors and ↓ affiliative behaviors; ↓ affiliative behaviors in dogs with higher scores on stranger-directed fear.
Colussi et al. [15]	90 min	Group	N/a	↓ Salivary cortisol after AAA compared to before session and home levels.
Uccheddu et al. [16]	30 min	Group	Weekly	↑ Salivary cortisol pre- and during session in one dog; most prevalent stress behaviors were tail down and lip licking.
Corsetti et al. [17]	10–30 min	Single	Max. 4 sessions/day with a 2 h break between sessions	No difference in anxiety-related or submissive behavior during AAIs; ↑ (non-aggressive) dominance behavior, attention, sniffing, affiliative and playful behavior.
Clark et al. [18]	15 min ± 5 SD	Group	Biweekly, weekly, 2/month, 1/month	↓ Salivary cortisol in biweekly sessions; one dog had higher post-session values in all conditions except the weekly session interval conditions; one dog had lower post-session values in all conditions except the biweekly session interval conditions.
Silas et al. [19]	90 min	Group	N/a	Self-reported handler (not client!) stress level was related to more stress behaviors in dogs; ↓ stress levels in students and dog handlers; ↑ stress when dog working levels were compared to baseline values at home.
Melco et al. [20]	60 min	Group	Biweekly	No changes in salivary cortisol, heart rate or stress-related behaviors emerged over 6 sessions; most prevalent stress behaviors were ears back, panting and lip licking.
De Carvalho et al. [21]	41.6 min ± 7.5 SD	N/a	Variable (ranging between 15 min to ≥24h)	↑ Salivary cortisol, heart rate and respiratory rate when working levels were compared to home baseline values; ↑ heart rates in AAA compared to AAT; ↑ heart and respiratory rate when dogs were transported >50 min.
Clark et al. [22]	<60 min	Single, Group	N/a	No difference in salivary cortisol comparing baseline values at home and working levels across multiple measurements; significant correlation between owner’s perceived stress in the dog and dog salivary cortisol; most prevalent stress behaviors were panting, lip licking and yawning.
Clark et al. [23]	20 min	Single	N/a	↓ Heart rate and right tympanic membrane temperature post session; no changes in cortisol, oxytocin or heart rate variability.
D′Angelo et al. [24]	70 min	Group of two inmates	Weekly	↓ Baseline salivary cortisol from pre- to post-program ↑ salivary cortisol during transportation.
